# Cell and Gene Therapy for Patients Suffering from Xerostomia (Dry Mouth): Positioning Extracellular Vesicles as the Bridge Between Biomarker Discovery and Regenerative Therapy in Xerostomia—A Scoping Review

**DOI:** 10.3390/ijms27135926

**Published:** 2026-06-30

**Authors:** Kumud Gogna, Hiba Mohammed Ali, Albert Leung, Shahnawaz Khijmatgar

**Affiliations:** 1 Department of Oral and Maxillofacial Surgery, University Hospital Limerick, Limerick V94 F858, Ireland; kumudgogna16@gmail.com; 2 University of Science and Technology, Omdurman, Sudan; hiba8019@gmail.com; 3 School of Dentistry, Royal College of Surgeons in Ireland, Dublin D18 NY72, Ireland; albertleung@rcsi.com

**Keywords:** extracellular vesicles, exosomes, xerostomia, salivary gland dysfunction, Sjögren’s syndrome, regenerative medicine, biomarker

## Abstract

Xerostomia is a common and debilitating condition caused by salivary gland dysfunction, frequently associated with primary Sjögren’s syndrome and head and neck radiotherapy. Current management is largely symptomatic and does not address underlying glandular injury. Extracellular vesicles (EVs), including exosomes, have emerged as candidate mediators of intercellular communication that have been proposed for diagnostic and therapeutic applications; however, their translational relevance to xerostomia remains uncertain and is currently supported only by exploratory evidence. This scoping review aimed to map and interpret current evidence on EV-based approaches in xerostomia and salivary gland dysfunction. A scoping review was conducted in accordance with “Preferred Reporting Items for Systematic Reviews and Meta-Analyses extension for Scoping Reviews (PRISMA-ScR)” checklist. Twenty-five articles were included, comprising 14 primary studies and 11 review articles. Studies were analysed based on application focus, methodological characteristics, reported outcomes, and translational readiness. Most primary studies focused on EVs as diagnostic biomarkers or their roles in immune–epithelial signalling. Therapeutic research was limited and largely confined to human-relevant translational models, namely human peripheral blood mononuclear cell (PBMC) assays and freshly resected human salivary gland tissue-maintained ex vivo. Outcomes were predominantly molecular and cellular, with minimal assessment of salivary flow or patient-reported symptoms. The current evidence base, although biologically plausible, remains exploratory: most included studies are mechanistic, and no clinical efficacy studies in xerostomia were identified. A substantial gap therefore persists between molecular findings and clinically meaningful outcomes, and further translational research is required before any conclusions can be drawn regarding the clinical utility of EV-based approaches.

## 1. Introduction

Before considering the molecular and cellular biology of xerostomia and its candidate therapies, it is important to distinguish three related but non-synonymous terms that are frequently used interchangeably in the literature. Xerostomia refers to the subjective sensation of oral dryness reported by the patient; it is a symptom and may occur with or without measurable changes in salivary output. Salivary hypofunction (or hyposalivation) refers to an objective, quantitatively measurable reduction in salivary flow rate, conventionally defined as an unstimulated whole-saliva flow rate of ≤0.1 mL/min or a stimulated whole-saliva flow rate of ≤0.7 mL/min. Salivary gland dysfunction is the broader pathophysiological concept that encompasses any structural, secretory, or compositional disturbance of the salivary glands, of which hyposalivation is one possible manifestation. These terms are not concordant: a patient may report xerostomia in the presence of normal flow rates (e.g., where saliva composition is altered), and conversely, measurable hyposalivation may exist without symptomatic complaint. Throughout this review, we use xerostomia for the patient-reported symptom, salivary hypofunction for the measured flow-rate deficit, and salivary gland dysfunction for the underlying pathophysiology, retaining the original terminology of each cited study where relevant.

Xerostomia significantly impairs oral physiology, taste perception, and overall quality of life by disrupting essential functions such as mastication, swallowing, speech, and oral mucosal protection [1,2]. Although commonly described as a subjective sensation of oral dryness, xerostomia often reflects underlying salivary gland dysfunction or structural damage [3]. This condition may arise from primary Sjögren’s syndrome, head and neck radiotherapy, metabolic disorders, or medication use, all of which reduce salivary secretion and contribute to persistent oral dryness [4].

Despite its substantial clinical burden, xerostomia has traditionally been managed by symptomatic relief rather than as a disorder rooted in salivary gland biology. This condition is highly prevalent worldwide, affecting approximately 20–30% of the general population. However, markedly higher rates are seen among older adults and individuals exposed to polypharmacy [5,6]. The prevalence exceeds 70–90% in patients receiving head and neck radiotherapy and approaches 90–100% among individuals with primary Sjögren’s syndrome [7,8]. The growing number of cancer survivors, ageing populations, and the increasing prevalence of chronic systemic diseases have collectively intensified the global burden of xerostomia, highlighting its significance as a public health challenge [6].

Current management strategies largely focus on symptomatic relief through saliva substitutes, topical lubricants, systemic sialagogues such as pilocarpine or cevimeline, and behavioural modifications. Although these approaches may provide temporary improvement, their clinical effectiveness is often limited by short duration of action, adverse systemic effects, poor adherence, and reduced benefit in cases involving irreversible glandular injury [9,10]. Importantly, these therapies do not address the inflammatory, fibrotic, or degenerative mechanisms underlying salivary gland dysfunction [11]. This therapeutic limitation reflects a long-standing assumption that salivary gland damage is irreversible, thereby constraining the development of restorative or regenerative treatment strategies.

Two cell- and gene-based modalities have entered early human evaluation for xerostomia. Adeno-associated virus serotype 2 encoding human aquaporin-1 (AAV2-hAQP1) progressed from a Phase 1 trial in irradiated parotid glands [12] through the AQUAx [13] and randomised Phase 2 AQUAx2 trials [14], with long-term follow-up reported separately [15]; however, viral immunogenicity, irreversible gene expression, and pre-existing neutralising antibodies remain key limitations. In parallel, mesenchymal stem cell (MSC) therapy has shown 30–50% salivary flow gains in radiation-induced xerostomia [16] and is being expanded in the MESRIX-more [17], University of Wisconsin AdMSC [18], and adipose-derived ASSIX [19] trials in Sjögren’s disease, though it carries the manufacturing and immunogenicity burdens of cell products. Against this backdrop, extracellular vesicles particularly exosomes are increasingly framed as a cell-free alternative, motivating the present scoping review.

Advances in regenerative medicine have begun to challenge this concept, suggesting that restoration of salivary gland function may be achievable through biological signalling mechanisms rather than direct cellular replacement [6]. Within this context, exosomes have emerged as candidate mediators of intercellular communication [20,21]. These extracellular vesicles (EV’s) transport proteins, lipids, and nucleic acids that modulate recipient cell behaviour. The surrounding lipid bilayer preserves molecular stability and supports biological activity [22,23].

In salivary research, exosomes have been extensively investigated as diagnostic biomarkers due to their stability and accessibility in biological fluids. However, their therapeutic potential for restoring salivary gland function remains insufficiently characterised [24,25]. There is some uncertainty regarding how EV-mediated signalling may influence inflammation, tissue repair, and functional recovery within damaged salivary glands.

Exosomes derived from mesenchymal stem cells (MSC’s) have attracted particular attention because they appear to retain many regenerative properties of their parent cells while avoiding risks associated with cell transplantation [24,25]. Preliminary experimental data, derived predominantly from in vitro and ex vivo models, suggest that these vesicles may modulate inflammatory responses and influence markers of tissue repair under controlled conditions. However, no clinical efficacy studies have yet been completed in xerostomia, and the prospect of moving from symptomatic management towards biological restoration therefore remains hypothetical and supported only by exploratory preclinical evidence [26,27,28].

Although recent narrative reviews have summarised extracellular vesicles in salivary research, most have focused predominantly on diagnostic applications and biomarker discovery. Therapeutic investigations remain scattered across heterogeneous experimental models and lack structured synthesis [29]. Up until now, no scoping review has systematically mapped exosome-based interventions for xerostomia with respect to study design, delivery approaches, functional salivary outcomes, and translational relevance.

Therefore, the present scoping review aims to map existing evidence on exosome-based approaches for the management of xerostomia, focusing on experimental context, therapeutic rationale, diagnostic applications, and reported outcomes. By identifying current knowledge gaps and research trends, this review aims to facilitate more coherent and clinically translatable strategies for salivary gland regeneration.

## 2. Materials and Methods

### 2.1. Study Design

This scoping review was conducted in accordance with the Preferred Reporting Items for Systematic Reviews and Meta-Analyses extension for Scoping Reviews (PRISMA-ScR) guidelines.

### 2.2. Protocol and Registration

The review protocol was registered on the Open Science Framework (OSF) platform https://doi.org/10.17605/OSF.IO/VUX7Z. Any deviations from the original protocol were documented and reported during the review process.

### 2.3. Eligibility Criteria

To address heterogeneity in study designs across the EV literature, eligibility was structured around three a priori tiers, each with explicit inclusion rules. This tiered framework allowed transparent integration of human clinical evidence, human-relevant translational evidence, and review/contextual evidence without conflating their respective weights.

Tier 1: Primary human studies. Studies enrolled human participants and reported EV-related diagnostic, mechanistic, or therapeutic data. Eligible designs included randomized and non-randomized clinical trials, observational and cross-sectional studies, case series, and case reports. Participants of any age or sex were eligible regardless of xerostomia aetiology (primary Sjögren’s syndrome, radiotherapy-induced injury, medication-related, systemic disease, or idiopathic).

Tier 2: Human-relevant translational evidence. Studies in which EVs of human origin were evaluated using human biospecimens (peripheral blood mononuclear cells, salivary biofluids) or freshly resected human salivary gland tissue (ex vivo). Studies of this tier were retained because they preserve biological relevance to human salivary gland physiology, and were analysed separately from Tier 1 evidence; they were not used to draw clinical conclusions.

Tier 3: Review articles for contextual synthesis only. Narrative, systematic, and scoping reviews on EVs in salivary gland disease were retained for contextual mapping of the field. Reviews did not contribute to outcome synthesis or to any conclusion regarding clinical or translational performance.

Studies were excluded if they used animal models without human-derived EVs or human tissue, in vitro investigations limited to immortalised cell lines or organoid models without human relevance, non-English publications without an available English full text, conference abstracts lacking full text, or studies not specifically focused on exosomes/EVs. Non-peer-reviewed sources (preprints) were retained only when they provided otherwise irreplaceable human-relevant translational data and were explicitly flagged as preprints in the synthesis, with the associated uncertainty acknowledged in the discussion and limitations.

### 2.4. Information Sources and Search Strategy

A comprehensive literature search was performed in PubMed, MEDLINE, Embase, Scopus, and Web of Science from database inception up to 1 January 2026. The search strategy combined controlled vocabulary terms (e.g., cyMedical Subject Headings (MeSH)) and free-text keywords related to extracellular vesicles (exosomes, extracellular vesicles, small extracellular vesicles, microvesicles, EVs) and salivary gland dysfunction (xerostomia, dry mouth, hyposalivation, salivary gland hypofunction, salivary secretion, and salivary gland diseases). Reference lists of included articles were additionally screened to identify relevant studies not captured through database searches.

### 2.5. Study Selection

**Figure 1:**
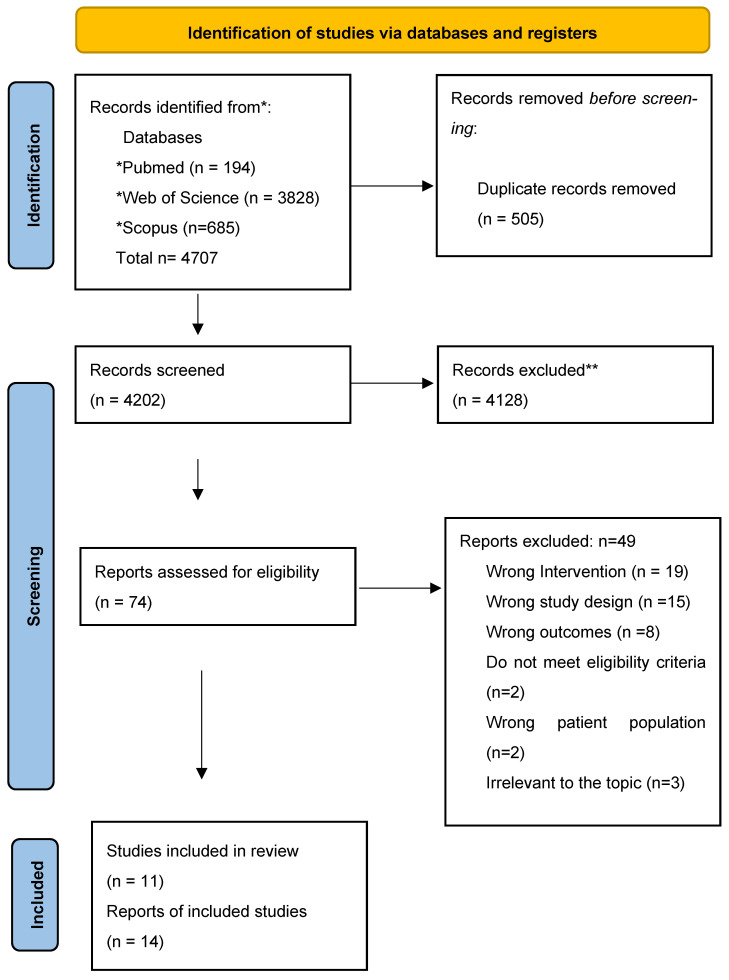
Preferred Reporting Items for Systematic Reviews and Meta-Analyses (PRISMA) 2020. * Consider, if feasible to do so, reporting the number of records identified from each database or register searched (rather than the total number across all databases/registers). ** If automation tools were used, indicate how many records were excluded by a human and how many were excluded by automation tools.

All identified records were imported into Covidence reference management software, and duplicates were removed. Titles and abstracts were screened independently by two reviewers, followed by full-text assessment of potentially eligible studies. Disagreements were resolved through discussion, with the consultation of a third reviewer when necessary. The study selection process is illustrated using a PRISMA flow diagram (Figure 1).

The numerical flow of selection (Figure 1) is as follows. A total of 4707 records were identified through database searching. Before screening, 505 records were removed: 464 duplicates and 41 records flagged as ineligible record types by automation tools (e.g., conference programmes, retracted records). This left 4202 records that proceeded to title-and-abstract screening, of which 4128 were excluded for not addressing extracellular vesicles in the context of salivary gland dysfunction. The remaining 74 full-text reports were assessed against the three-tier eligibility framework described in Section 2.3. Of these 74 reports, 49 were excluded with documented reasons: wrong intervention (n = 19), wrong study design (n = 16), wrong outcome (n = 9), non-English full text not available (n = 3), and other reasons such as data unretrievable from the original authors (n = 2). The final 25 articles meeting eligibility were classified as 14 primary studies (Tiers 1–2) and 11 review articles (Tier 3, retained for contextual synthesis only). All quantitative descriptors reported in the Results Section are based exclusively on the 14 primary studies.

### 2.6. Data Charting and Data Items

Data extraction was performed using a standardized charting form developed and piloted prior to data collection. Two reviewers independently extracted data, with discrepancies resolved by consensus. Extracted information included study characteristics, exosome source and processing methods, application focus (diagnostic, exploratory or therapeutic), outcomes assessed, key findings and reported translational relevance.

Compliance with Minimal Information for Studies of Extracellular Vesicles (MISEV) guidelines was recorded where available. For review articles, data extraction focused on review scope, discussed applications of EVs, methodological challenges, major conclusions, and identified research gaps.

### 2.7. Data Synthesis

Given the purpose of this scoping review, findings were synthesised descriptively. Studies were categorised using a diagnostic, exploratory and therapeutic framework to distinguish therapeutic or regenerative applications. Outcomes were grouped into four predefined domains: salivary flow measurements, xerostomia symptom outcomes, salivary gland functional or structural outcomes and biomarker-related outcomes.

A MISEV-informed heat map was generated to summarise reporting of EV source, isolation, processing, and characterisation among primary studies [30]. Therapeutic studies were further mapped using a translational readiness framework to describe progression from exploratory research towards clinically relevant human investigations. Technological Readiness Levels (TRLs) were adapted as a conceptual framework to assess translational maturity. Review articles were synthesised narratively to identify methodological challenges, research gaps, and future directions.

### 2.8. Critical Appraisal

Consistent with the methodological frameworks of Arksey and O’Malley (2005) [31] and the Joanna Briggs Institute (JBI) for scoping reviews, a formal quantitative risk-of-bias appraisal of individual studies was not undertaken; the purpose of a scoping review is to map the breadth and structure of an evidence base rather than to grade the certainty of effect estimates. To still provide the reader with an indication of evidence maturity, every included study was assigned a level of evidence using the Johns Hopkins Evidence-Based Practice (JHEBP) hierarchy, and therapeutic studies were positioned on a Technology Readiness Level (TRL) framework adapted for biomedical translation. The acknowledged absence of formal risk-of-bias tooling is restated as a limitation in Section 4.3.

### 2.9. Ethics and Dissemination

Ethical approval was not required as this study analysed previously published literature. Findings will be disseminated through peer-reviewed publication and academic conference presentations. The current scoping review was presented at the Irish Society of Cell and Gene Therapy, Galway, Ireland as a poster presentation.

## 3. Results

### 3.1. Study Selection and Characteristics

A total of 25 articles met the inclusion criteria, including 14 primary research studies and 11 review articles. Most primary studies were cross-sectional observational investigations in human populations, with a small number of human-relevant experimental studies, including ex vivo models. Among the review articles, narrative reviews predominated, although a small number used systematic or scoping review approaches. A detailed summary of study characteristics is provided in Supplementary File S1.

Early studies published around 2010 primarily focused on the feasibility and reliability of isolating exosomes, establishing proof-of-concept for extracellular vesicle extraction. From approximately 2013 onward, research expanded to investigate EVs as potential diagnostic biomarkers, alongside increasing variation in isolation techniques and growing clinical interest.

From 2016 onward, studies increasingly incorporated ex vivo experiments and immunomodulatory assays, reflecting a shift toward investigating the biological functions of EVs rather than simply detecting their presence. However, relatively few studies explored their therapeutic potential in clinical contexts. Most research contributions originated from China and several European centres, particularly Norway, Italy, and Greece, with fewer studies conducted in the United States.

The 14 primary studies were classified according to their main application domain. Seven studies investigated EVs as diagnostic biomarkers, five explored biological mechanisms, and two examined potential therapeutic applications. Diagnostic studies relied almost entirely on cross-sectional human observational designs. Exploratory studies typically combined human biospecimens with in vitro or ex vivo functional assays to explore immune-epithelial pathways. Therapeutic investigations were limited, with only two studies identified, including one using a human ex vivo salivary gland irradiation injury model.

### 3.2. Application Focus of Included Primary Studies

#### 3.2.1. Diagnostic Biomarker Studies

Diagnostic investigations were predominantly cross-sectional studies analysing EVs isolated from saliva, plasma, or tear fluid. These studies aimed to identify molecular signatures associated with primary Sjögren’s syndrome, sicca conditions, or related autoimmune diseases, often comparing affected individuals with healthy controls or disease comparators such as rheumatoid arthritis or systemic lupus erythematosus.

Reported outcomes were primarily molecular or omics-based, including differential expression of proteins, microRNAs, circular RNAs, and other RNA species. However, none of the diagnostic studies incorporated clinically meaningful xerostomia outcomes. Objective measures such as unstimulated or stimulated salivary flow were not used as primary outcome measures, and validated xerostomia symptom questionnaires or quality-of-life instruments were not reported. When salivary flow was recorded, it was typically used only to confirm eligibility or sample adequacy rather than to assess glandular function.

As a result, diagnostic findings largely remained at the level of molecular discrimination between study groups, without direct confirmation that identified EV signatures correspond to functional salivary gland impairment.

#### 3.2.2. Exploratory/Biological Studies

Exploratory studies examined the potential biological role of EVs in salivary gland dysfunction. These investigations integrated human-derived samples with in vitro or ex vivo experimental models to evaluate how EVs influence cellular pathways involved in glandular homeostasis.

Several studies explored immune–epithelial communication, examining how EVs modify intracellular signalling processes within salivary gland epithelial cells [31,32,33,34]. Mechanisms investigated included alterations in calcium and cyclic AMP (cAMP) signalling pathways, oxidative stress responses, ferroptosis-related cell injury, and abnormal activation of immune cells.

Outcomes were generally restricted to cellular or molecular outcomes, such as intracellular Ca^2+^ flux, amylase secretion, changes in intracellular signalling pathways, and markers of epithelial injury. While these findings provided biological insight, they were not linked to functional salivary gland outcomes in human populations.

#### 3.2.3. Therapeutic Studies

Therapeutic studies were few in number and largely confined to human-relevant translational models. Two therapeutic primary studies were identified [35,36]: one used human peripheral blood mononuclear cells (PBMCs) co-cultured with human bone-marrow MSC-derived exosomes to model T-cell subset balance and cytokine output in primary Sjögren’s syndrome [35], and the other used freshly resected human salivary gland tissue maintained ex vivo and exposed to ionising radiation, with subsequent treatment by human MSC-derived EVs [36]. In both studies the recipient cells/tissue and the EV source were of human origin; no animal tissue was used in the therapeutic readouts. It should be explicitly noted that the second of these therapeutic studies [36] is a preprint deposited on bioRxiv and, at the time of writing, has not undergone formal peer review. Its findings should therefore be interpreted with appropriate caution: the methodological details, statistical analyses, and biological conclusions reported in non-peer-reviewed sources have not been independently scrutinised, and may be subject to revision before formal publication. The preprint was retained in this scoping review because excluding it would have left only a single peer-reviewed therapeutic data point in the entire literature on EV-based xerostomia interventions, and because its content (human ex vivo salivary tissue model, full EV characterisation) is otherwise irreplaceable for mapping the therapeutic landscape. Readers are nonetheless encouraged to verify whether reference [36] has since been peer-reviewed before drawing further inferences.

Reported outcomes included improvements in immune balance, tissue viability, and markers of cellular injury. However, EV-based interventions were not evaluated in patients, and none of the studies demonstrated recovery of salivary flow or improvement in xerostomia symptoms.

**Figure 2:**
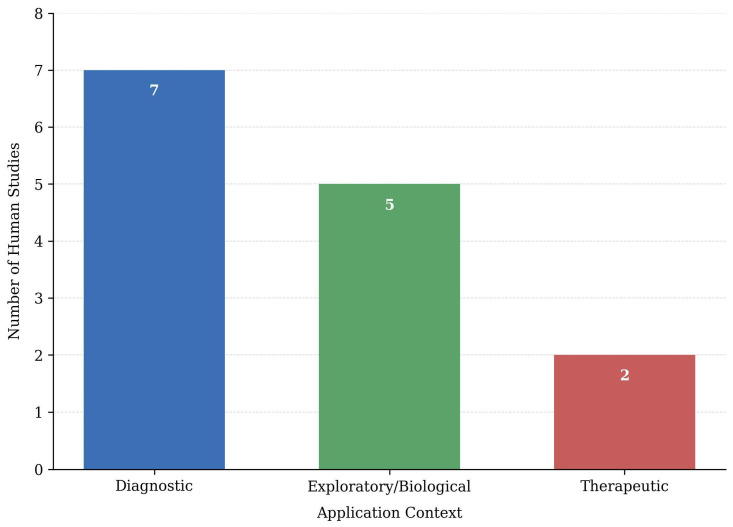
Distribution of human studies according to the application context of cell and gene therapy (G) in xerostomia. The bar chart illustrates the number of included human studies categorized by their primary application context. Diagnostic applications were the most frequently reported (n = 7), followed by exploratory/biological investigations (n = 5), while therapeutic applications were less commonly represented (n = 2).

Diagnostic research appeared more advanced than therapeutic investigations in terms of translational development. The distribution of the 14 primary human studies by application context—diagnostic (n = 7), exploratory/biological (n = 5), and therapeutic (n = 2) is illustrated in Figure 2.

### 3.3. Population and Clinical Context

Most studies investigated patients with primary Sjögren’s syndrome, reflecting the strong association between this condition and xerostomia. One study examined radiation-induced salivary gland injury. Several studies included healthy controls, while others incorporated autoimmune disease comparators such as rheumatoid arthritis or systemic lupus erythematosus.

Sex distribution was frequently skewed toward female participants, reflecting the epidemiology of Sjögren’s syndrome. Reporting of age and sex varied across studies, although some reported age- and sex-matched control groups. Diagnostic classification most commonly followed the 2016 American College of Rheumatology/European League Against Rheumatism (ACR/EULAR) criteria, while earlier studies used the American-European Consensus Group criteria.

### 3.4. EV Terminology, Biological Sources, and Methodological Heterogeneity

The terms “extracellular vesicles” and “exosomes” were often used interchangeably across studies, frequently without clear distinction based on vesicle size or biogenesis. Throughout this review, “extracellular vesicles (EVs)” is used as the umbrella term, while “exosomes” is reserved for the small-EV subtype (typically 30–150 nm; endosomal origin) where the original studies provided characterisation consistent with this definition, in line with MISEV2018/MISEV2023 recommendations.

EVs were isolated from a range of biological sources, including stimulated and unstimulated saliva samples, tear fluid, plasma or serum, cultured immune or stem cells, and ex vivo salivary gland tissues.

Isolation methods varied substantially and included ultracentrifugation, size-exclusion chromatography, precipitation-based kits, and proprietary isolation platforms. EV characterisation methods also differed widely. Some studies used multi-modal validation approaches, while others relied on minimal characterisation. This methodological variability limits direct comparison across studies.

### 3.5. EVs and Biological Pathways

The EV cargoes analysed varied according to study objectives and analytical platforms. Investigated molecules included proteins, microRNAs, circular RNAs, and broader EV RNA profiles such as tRNA-derived and long non-coding RNA species.

Diagnostic studies commonly associated EV signatures with immune and inflammatory pathways, including neutrophil activation, tumour necrosis factor/nuclear factor kappa B (TNF/NF-κB) signalling, and interferon-related responses. Exploratory studies demonstrated that EVs could influence salivary gland epithelial signalling, affecting Ca^2+^ signalling, aquaporin 5/stromal interaction molecule 1 (AQP5/STIM1) related pathways, and cAMP-dependent amylase secretion. Therapeutic studies reported in vitro and ex vivo signals consistent with possible immunomodulatory activity of mesenchymal stem cell-derived EVs, including changes in T-helper 17 cell/regulatory T cell (Th17/Treg) balance and in markers of tissue injury in irradiation models; these findings are preliminary and have not been confirmed in patients.

Across the literature, EVs were investigated in three main roles: as diagnostic biomarkers, mediators of glandular dysfunction, and potential therapeutic agents.

### 3.6. Minimal Information for Studies of Extracellular Vesicles (MISEV) Compliance

The Minimal Information for Studies of Extracellular Vesicles (MISEV) guidelines provided standardised reporting criteria for EV identification and characterisation.

**Figure 3:**
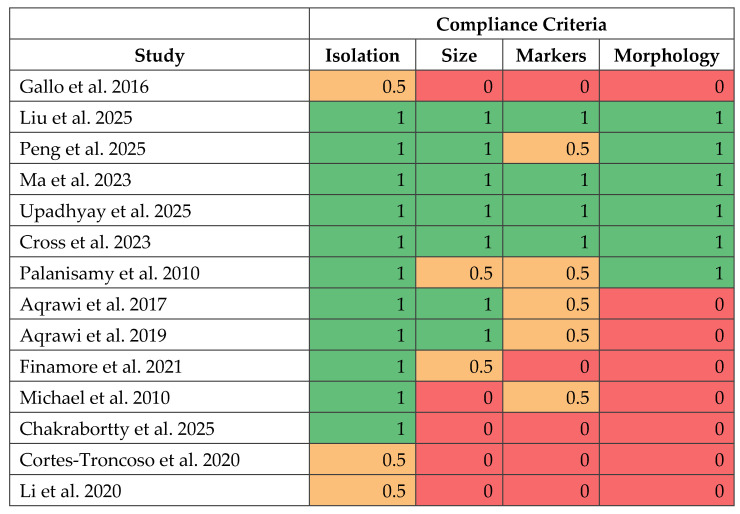
Illustrating Compliance Matrix Scores for studies included. Presenting a heat map of MISEV compliance across included studies and demonstrates variability in EV isolation, characterization and reporting [32,33,34,35,36,37,38,39,40,41,42,43,44,45].

Reporting quality was assessed across four key domains: EV isolation methods, particle size characterisation, EV marker profiling, and morphological validation. Compliance varied across the included studies (Figure 3).

Four studies demonstrated full compliance, reporting all four criteria. Three studies showed high but incomplete compliance, while six demonstrated partial or low compliance, most commonly due to missing EV marker profiling or morphological validation.

EV isolation methods were the most consistently reported methodological element, whereas marker profiling and morphological characterisation were frequently absent. Reporting of negative controls and cell-of-origin markers was also inconsistent, which might have a negative impact on reproducibility.

### 3.7. Translational Readiness of Primary Studies

Using the Technology Readiness Level (TRL) framework, most studies were classified at early translational stages.

The majority clustered at TRL 3 (experimental proof-of-concept), representing biomarker identification or methodological validation without clinical implementation. Two studies were classified as TRL 2, demonstrating feasibility of isolating EVs from saliva and detecting biologically active RNA.

Three studies reached TRL 4, providing experimental evidence linking EVs to epithelial injury or salivary gland dysfunction. Two studies reached TRL 5, using ex vivo human tissue or immune-cell models to evaluate EV-mediated mechanisms. Only one study reached TRL 6, reporting an association between EV-associated microRNAs and pathological CD4^+^ T-cell activation in humans.

No studies progressed to higher translational stages involving clinical testing of EV-based interventions (TRL 7–9) or demonstrated improvements in patient outcomes such as salivary flow or xerostomia symptoms.

The evidence base remains concentrated at TRL 2–3, indicating that most research is still focused on biomarker discovery and early proof-of-concept studies rather than clinical application (Table 1).

### 3.8. Contextual Synthesis from Included Review Articles

The 11 review articles [26,27,29,46,47,48,49,50,51,52,53] included in this scoping review are synthesised here separately from the primary evidence to avoid conflation. Reviews were used solely for contextual mapping of research gaps, methodological challenges, and translational barriers; they did not contribute to the outcome distributions, MISEV scoring, or TRL positioning reported in Section 3.1, Section 3.2, Section 3.3, Section 3.4, Section 3.5, Section 3.6 and Section 3.7.

EV research in xerostomia-related conditions was consistently described as early-stage and methodologically diverse. Most reviews summarised observational biomarker studies and preclinical experiments, with few addressing clinical dry mouth outcomes. Common gaps included limited standardisation of EV isolation and saliva processing, inconsistent EV characterisation, and weak association between EV findings and clinically meaningful outcomes. Reviews also highlighted major barriers to translation, including lack of standardised EV manufacturing, uncertainty around dosing and delivery, limited safety data, and challenges in patient stratification and endpoint selection. Most included reviews were Level IX narrative reviews, with only a few Level III systematic or scoping reviews, indicating that the evidence base was largely exploratory and yet to be supported by high-level clinical evidence (Table 2) (Supplementary File S1).

## 4. Discussion

The aim of this scoping review was to map and interpret the current evidence on extracellular vesicles (EVs), including exosomes, in xerostomia and salivary gland dysfunction. Xerostomia is a common and clinically important condition associated with primary Sjögren’s syndrome, radiation-induced salivary gland injury, systemic autoimmune disease, and medication-related salivary hypofunction. These conditions significantly affect oral health, nutrition, and quality of life. Despite this burden, current treatment regimes remain largely symptomatic and do not restore salivary gland function. EVs have therefore attracted an increasing level of interest as potential diagnostic biomarkers and therapeutic agents. However, the extent to which this research has progressed toward clinical application remains unclear.

This review demonstrates that EV research in xerostomia is still largely concentrated in diagnostic biomarker discovery and biological investigation, with relatively limited exploration of therapeutic applications. Overall, this indicates that the field remains at an early stage of development, with limited progression toward clinical implementation.

A major finding of this review is the lack of clinically meaningful xerostomia outcomes across the evidence base. As a result, diagnostic studies were largely limited to identifying molecular differences between disease groups and controls.

Most diagnostic investigations also relied on cross-sectional observational designs, which allowed comparisons between groups but not assessments of disease progression or changes in salivary gland function over time. Consequently, current EV biomarkers remain at a proof-of-concept stage, and their clinical value for predicting disease severity or informing patient management remains guarded.

Exploratory studies provided useful insight into how EVs might contribute to salivary gland dysfunction. These studies showed that EVs can influence immune–epithelial communication and affect cellular processes relevant to salivary secretion. For example, EV-associated molecules were reported to disrupt calcium signalling, cyclic AMP-dependent secretion, and aquaporin-mediated water transport in epithelial cells. Other studies identified links with oxidative stress, ferroptosis, and immune activation [32,33,34,44]. However, these findings were mainly derived from cell culture or ex vivo experiments, and none of the studies assessed whether these mechanisms translate into measurable changes in salivary gland function in living patients.

Therapeutic investigations were particularly limited. The few available studies focused on mesenchymal stem cell-derived EVs in human-relevant translational models (human PBMC immune-cell assays and ex vivo human salivary gland tissue) [35,36]. These studies reported improvements in immune regulation, reduced cellular injury, and protective effects on salivary gland tissues. However, none of them evaluated EV-based therapies in human patients, and none demonstrated restoration of salivary secretion or improvement in xerostomia symptoms. Therapeutic EV research in xerostomia therefore remains preclinical.

Available evidence suggests that the limited clinical translation of EV research in xerostomia is not simply due to the absence of clinical trials. Rather, this field is currently weighted toward molecular discovery and biological exploration, with studies incorporating functional and patient-centred outcomes remaining few and far between. Advances in EV profiling, including multi-omics approaches applied to saliva, plasma, and tear fluid, have improved molecular characterisation. However, these advances have yet to be matched by equivalent progress in clinically relevant outcome assessment.

Despite these limitations, the available evidence supports a preliminary biological rationale for further investigation of EVs in xerostomia. Several studies identified EVs linked to immune and inflammatory pathways already known to be involved in salivary gland disease, particularly in primary Sjögren’s syndrome. Exploratory studies further showed that EV-associated molecules can interfere with pathways required for normal glandular secretion; however, these findings remain hypothesis-generating and have not been validated in clinical settings.

Among the therapeutic-oriented studies, two reports [35,36] represent important advances because they combined detailed EV characterisation with human-relevant immune and salivary gland models. Their findings provide early experimental signals consistent with possible immunomodulatory or cytoprotective activity of mesenchymal stem cell-derived exosomes under controlled conditions; these signals are based on cellular and tissue-level readouts and have not been confirmed in patients. An additional and important caution applies specifically to reference [36], which is a non-peer-reviewed preprint deposited on bioRxiv. The absence of formal peer review introduces further uncertainty about the methodology, statistical handling, and biological interpretation of its findings, and any inferences drawn from this single source should therefore be treated as tentative. This is particularly relevant given that reference [36] represents one of only two therapeutic data points in the entire EV/xerostomia literature; any future clinical translation effort that relies on its results would benefit from waiting until a peer-reviewed version is available, or from independent replication. However, the absence of in vivo functional outcomes highlights the continuing gap between findings and clinical application.

One possible explanation for this gap is the complex physiology of salivary secretion. Saliva production depends on coordinated interactions between epithelial cells, nerves, blood vessels, and ductal structures within the gland. In contrast, many EV studies focus on isolated molecular pathways or cellular responses. While these models provide important biological insight, they may not fully reflect the integrated processes required to restore glandular function.

Overall, the findings of this review suggest that EV research in xerostomia has advanced substantially at the molecular level. However, translation toward clinically meaningful benefit remains limited. Future studies should incorporate standardised xerostomia outcome measures, longitudinal designs, and functional assessments of salivary gland recovery. Addressing these gaps will be essential if EV-based discoveries are to contribute meaningfully to the diagnosis and treatment of xerostomia.

### 4.1. Integration with Existing Literature

Previous narrative and systematic reviews consistently describe extracellular vesicles, particularly exosomes, as biologically plausible diagnostic and therapeutic tools in salivary gland disease [49,53,54]. Most of this literature focuses on primary Sjögren’s syndrome and radiation-induced salivary gland injury. These reviews highlight the role of EVs in immune–epithelial communication, interferon-driven inflammation, calcium signalling, cyclic AMP regulation, aquaporin trafficking, epithelial stress responses, and regenerative signalling [53,54]. The findings of the present scoping review are broadly consistent with this framework.

Several reviews have specifically emphasised the diagnostic potential of EVs [29,54]. These studies highlight the stability of EVs in saliva, serum, and tear fluid, as well as their ability to carry disease-related proteins and nucleic acids relevant to the early detection of primary Sjögren’s syndrome. Our findings support these observations and confirm that diagnostic biomarker discovery remains the dominant focus within the field [29,49,52,53,54]. However, by mapping study intent and outcome selection, this present review further shows that most diagnostic investigations remain limited to cross-sectional comparison and early proof-of-concept findings, with little evidence of prospective validation or clinically actionable thresholds.

In addition to diagnostics, several narrative reviews discuss the therapeutic promise of mesenchymal stem cell-derived exosomes [29,49]. These studies emphasise their immunomodulatory, cytoprotective, and regenerative properties, as well as their potential advantages over live-cell therapies, including lower immunogenicity and improved safety profiles [29,53]. While the present review acknowledges this biological rationale, it also shows that the available therapeutic evidence remains preclinical, with no current evidence of sustained functional recovery of salivary gland secretion in patients.

Reviews focusing on regenerative medicine place exosome-based strategies within a broader therapeutic landscape that includes stem cell transplantation, secretome-based approaches, gene therapy, and tissue engineering [52,53]. These reviews report improvements in salivary flow and glandular architecture in animal models following stem cell- or exosome-based interventions [52,53]. However, these findings are mainly derived from preclinical studies and short-term experimental outcomes.

The present review extends this literature by shifting the emphasis from biological plausibility alone to the translational structure of the evidence base. Rather than attributing the limited clinical translation only to a lack of late-phase trials, this study’s synthesis identifies a broader issue: across diagnostic, exploratory, and therapeutic studies, clinically meaningful xerostomia outcomes are rarely incorporated. Salivary flow rates, validated symptom instruments, and patient-reported quality-of-life measures are largely absent. This suggests a probable disconnect between molecular or cellular outcomes and the clinical features used to define xerostomia.

Importantly, this issue has not been addressed systematically in earlier reviews. Previous syntheses provide comprehensive summaries of EV biology and preclinical therapeutic effects, but they rarely examine how outcome selection influences translational relevance. By mapping studies according to research intent, outcome measures, and translational stage, the present review helps explain why promising EV findings have yet to be translated into measurable patient-level benefit.

Interest in EV-based therapeutics is also reflected in the wider cell and gene therapy community. For example, sessions dedicated to EV biology, manufacturing, and clinical translation were included in the 2026 annual meeting of the Irish Society for Gene and Cell Therapy (ISGCT) in Galway. These discussions highlighted growing interest in EVs as a research platform, while also emphasising challenges related to manufacturing, standardisation, and clinical translation. This broader context is consistent with the findings of the present review, which identify continued research interest in EV biology but limited progression toward clinically validated therapies.

Overall, both the existing literature and the present scoping review suggest that EVs are a biologically plausible candidate platform for further investigation in xerostomia-related salivary gland dysfunction, recognising that current evidence does not yet support claims of therapeutic effect. However, the evidence base remains heavily focused on molecular mechanisms and short-term tissue outcomes. Greater integration of functional salivary gland measures and patient-centred outcomes will be necessary to support meaningful clinical translation.

### 4.2. Limitations of the Included Evidence

The evidence identified in this review has several notable limitations. Most primary studies used cross-sectional observational designs or preclinical experimental models, limiting insight into disease progression, treatment efficacy, and long-term outcomes. Human studies were mainly restricted to diagnostic sampling and rarely incorporated functional salivary measures or patient-reported outcomes. As a result, although knowledge is increasing, clinical interpretation remains limited. A further methodological limitation is that, consistent with scoping-review methodology, no formal risk-of-bias appraisal of individual studies was conducted; readers should therefore interpret the comparative pattern of findings descriptively rather than as evidence of effect certainty. Future systematic reviews of specific EV applications (e.g., diagnostic accuracy in primary Sjögren’s syndrome, MSC-EV efficacy in radiation xerostomia) would benefit from formal risk-of-bias assessment using design-appropriate tools (QUADAS-2, RoB 2, ROBINS-I, OHAT, or SYRCLE).

A further specific limitation concerns the status of the published source material. One of the two therapeutic primary studies included in this review [36] is a preprint that has not undergone formal peer review at the time of writing. Although it was retained because excluding it would have left only a single therapeutic data point in the entire EV/xerostomia literature and because its methodological scope (full EV characterisation in freshly resected human salivary gland tissue-maintained ex vivo) is otherwise unmatched, its non-peer-reviewed status introduces an additional layer of uncertainty into the therapeutic evidence base. The reported findings, the statistical handling, and the biological inferences of reference [36] have not yet been independently scrutinised by external referees, and any subsequent peer-reviewed version may differ from the preprint in important respects. We therefore explicitly flag this reference as a preprint throughout the manuscript and recommend that future syntheses revisit this evidence once a peer-reviewed publication is available.

Methodological heterogeneity was also common. Studies differed in EV source, saliva collection protocols, isolation methods, and characterisation strategies. Adherence to MISEV reporting guidelines was inconsistent, particularly in relation to EV marker profiling and morphological validation. These issues reduce reproducibility and make comparison between studies more difficult. Although studies published following the 2018 MISEV update reported more frequently key EV characterisation parameters, compliance remained variable [41].

Incomplete EV characterisation also affects interpretation of biological findings. When vesicle purity or cellular origin is uncertain, reported biological effects may not be attributable specifically to EVs. Molecules described as “exosomal” may instead reflect co-isolated proteins, lipoproteins, or mixed vesicle populations. In such cases, uncertainty in EV identity weakens confidence in the reported biological associations.

### 4.3. Implications for Clinical Practice and Research

#### 4.3.1. Clinical Practice Application

At present, EV-based diagnostics and therapies for xerostomia are not ready for routine clinical use [55,56]. Although early diagnostic potential has been reported, substantial work is still required before clinical implementation can be considered. This includes prospective validation of EV biomarkers in well-characterised patient cohorts, standardisation of EV isolation and analysis methods, and development of regulatory frameworks suitable for saliva-based diagnostic technologies [55,56].

Therapeutic translation presents additional challenges. These include the need for reproducible GMP-grade EV production, identification of appropriate dosing and delivery strategies, comprehensive safety evaluation, and regulatory classification of EV-based products [57,58]. EV-based therapeutic approaches should therefore currently be regarded only as investigational.

The cost structure of extracellular vesicle (EV) therapeutics is currently driven predominantly by upstream and downstream biomanufacturing processes rather than by the EV product itself. Clinical-grade production requires GMP-compliant parental MSC banking, qualification of xeno-free and chemically defined culture media, scalable isolation workflows such as tangential-flow filtration (TFF) combined with size-exclusion chromatography (SEC), and extensive release-quality control assays, including sterility, endotoxin testing, nanoparticle tracking analysis (NTA), EV marker profiling, identity, purity, and potency assessment. Collectively, these manufacturing and quality-assurance requirements impose substantial translational costs that may approach those associated with advanced cell therapy products under low-scale or patient-specific production models. Until robust allogeneic, off-the-shelf EV platforms with validated potency, reproducibility, and long-term storage stability are established, EV-based therapies for xerostomia are unlikely to achieve cost competitiveness with current symptomatic management approaches on a per-patient basis.

#### 4.3.2. Future Research

Future research should focus on prospective human studies that include clinically meaningful outcomes. These should incorporate objective measurements of salivary flow alongside validated xerostomia symptoms and quality-of-life instruments [9,59]. Development in this area will require stronger links between molecular findings and patient-level outcomes. Diagnostic, exploratory, and therapeutic studies should therefore be designed in a complementary way rather than remaining isolated at molecular or cellular levels.

Standardisation will be an important factor in effecting meaningful further progress. Harmonisation of EV isolation, characterisation, and pre-analytical saliva handling protocols will be highly desirable to enhance reproducibility and comparability across different studies [55]. In parallel, therapeutic research should also move towards carefully designed human interventional studies supported by dose-finding, delivery optimisation, and safety assessment [57,58].

Stratification of patients by disease characteristics may also be important. Factors such as serological status, disease stage, and residual salivary gland function may influence treatment response and should be considered in future study design [59].

Within the broader cell and gene therapy landscape, exosome-based therapies are best regarded as a candidate cell-free strategy still under investigation for salivary gland dysfunction [60]. However, progress toward clinical translation will depend not only on biological efficacy but also on advances in manufacturing and delivery technologies. Key challenges include scalable EV production, definition of critical quality attributes, reproducible purification methods, and optimisation of delivery strategies to ensure EV retention and activity within salivary gland tissue [58].

Finally, translational progress should be judged not only by technological complexity but also by clinical relevance. Studies that do not include functional and patient-centred endpoints remain limited in translational value, regardless of their molecular depth [40,45]. Addressing these challenges will require closer collaboration between oral medicine, immunology, regenerative medicine, bioengineering, and regulatory science [56]. In this context, adherence to harmonised MISEV-compliant methodologies should be regarded as essential for producing reliable and interpretable human research [55].

## 5. Conclusions

This scoping review demonstrates that research on extracellular vesicles (EVs) in xerostomia has progressed considerably at the molecular and biological levels but has yet to be translated into clinical application. The current evidence base is dominated by early to mid-translational studies, considerable methodological heterogeneity, and a consistent lack of outcomes that directly reflect salivary gland function or patient experience.

Although EVs demonstrate biological plausibility as both diagnostic biomarkers and therapeutic tools, limited methodological standardisation and the absence of clinically relevant endpoints restrict the interpretation and translational value of existing studies. It is important to emphasise that no clinical efficacy studies of EV-based interventions in xerostomia were identified, and the conclusions of this review should therefore be interpreted as a description of an exploratory and largely mechanistic literature rather than as evidence supporting therapeutic use. Without study designs that incorporate functional outcomes and clearly defined EV products, research risks become increasingly detailed at the molecular level while remaining clinically uninformative. Future studies should focus on outcome-oriented research that integrates objective salivary flow measurements, validated xerostomia symptom scales, and patient-reported quality-of-life outcomes.

## Figures and Tables

**Table 1:** Technology Readiness Levels (TRLs) of included primary studies (TRL 2–6).

TRL	Description	Included Studies	Findings/Translational Significance
TRL 2	Exploratory discovery/feasibility	Michael et al. 2010 [42]; Palanisamy et al.2010 [38]	Feasibility of salivary EV profiling; structural and RNA characterisation; demonstration of EV-mediated RNA transfer in saliva
TRL 3	Analytical biomarker proof-of-concept	Aqrawi et al. 2017 [39], 2019 [40]; Li et al. 2020 [45]; Finamore et al. 2021 [41]; Cross et al. 2023 [37]; Chakrabortty et al. 2025 [43]	Salivary, tear, and plasma EV signatures distinguishing Sjögren’s disease; recurrent inflammatory markers (LCN2, S100A8/A9, ANXA2, CD14); EV circRNAs (circ-IQGAP2, circ-ZC3H6); tRNA fragments; long-RNA profiles stratifying SSA^+^ vs. SSA^−^
TRL 4	Preclinical functional/mechanistic validation	Gallo et al. 2016 [32]; Cortes-Troncoso et al. 2020 [44]; Peng et al. 2025 [34]	EV miRNAs impair epithelial Ca^2+^/cAMP signalling (miR-BART13-3p, miR-142-3p); EV ferroptosis-related proteins linked to epithelial injury
TRL 5	Advanced preclinical/human ex vivo evidence	Ma et al. 2023 [35]; Upadhyay et al. 2025 [36]	MSC-derived EVs restore Th17/Treg balance, modulate autophagy, and reduce salivary gland injury (Ma et al. 2023 [35]: human PBMC immune-cell assay with human bone-marrow MSC-derived exosomes; Upadhyay et al. 2025 [36]: freshly resected human salivary gland tissue-maintained ex vivo with human MSC-derived EVs). All therapeutic readouts in the included primary studies use human PBMCs or human ex vivo salivary tissue; no rodent tissue is used in the therapeutic outcomes.
TRL 6	Early translational human evidence	Liu et al. 2025 [33]	Plasma-derived EV miR-501-3p promotes CD4^+^ T-cell activation and Tfh differentiation in Sjögren’s disease patients

*Note*: ANXA2, annexin A2; Ca^2+^, calcium ion; cAMP, cyclic adenosine monophosphate; CD4, cluster of differentiation 4; CD14, cluster of differentiation 14; circ-IQGAP2, circular RNA derived from the IQ motif-containing GTPase activating protein 2 gene; circ-ZC3H6, circular RNA derived from the zinc finger CCCH-type containing 6 gene; circRNA, circular RNA; EV, extracellular vesicle; LCN2, lipocalin-2; miR-142-3p, microRNA-142-3p; miR-501-3p, microRNA-501-3p; miR-BART13-3p, Epstein–Barr virus-encoded microRNA BART13-3p; miRNA/miR, microRNA; MSC, mesenchymal stem cell; PBMC, peripheral blood mononuclear cell; RNA, ribonucleic acid; S100A8/A9, S100 calcium-binding proteins A8 and A9; SSA, Sjögren’s syndrome A antigen (anti-Ro autoantibody); Tfh, T follicular helper cell; Th17, T-helper 17 cell; Treg, regulatory T cell; tRNA, transfer RNA.

**Table 2:** Translational challenges in EV-based xerostomia research (synthesised from the 11 included review articles; contextual reference only).

Domain	What Reviews Report	Why This Matters for Translation
Clinical outcome gap	Few studies link EV findings to salivary flow, dry mouth symptoms, or quality of life; little long-term follow-up	Makes it difficult to judge real clinical benefit
Diagnostic validation gap	Biomarkers proposed, but rarely tested in large or prospective patient cohorts	EV diagnostics remain experimental
Therapeutic evidence gap	Most therapeutic studies are human ex vivo or human cell-based; no robust human in vivo trials	Effectiveness and safety in patients are unknown
EV isolation variability	Different isolation methods used across studies	Results cannot be easily compared
Pre-analytical variability	Differences in saliva type and sample handling	Reduces reproducibility
EV characterisation inconsistency	Many studies do not fully follow MISEV guidelines	Low confidence in EV purity and identity
EV source diversity	EVs taken from saliva, blood, tears, immune cells, or stem cells	No clear best source for diagnosis or treatment
Disease heterogeneity	Patient subtypes and disease stages often not separated	Important subgroup effects may be missed
Manufacturing challenges	No standardised method to produce clinical-grade EVs	Limits progression to human trials
Regulatory uncertainty	EVs do not fit clearly into existing drug categories	Slows trial approval
Outcome measure inconsistency	Different studies use different endpoints	Prevents data comparison and synthesis
Safety/immunogenicity	Allogeneic MSC-derived EVs carry surface HLA antigens that may trigger T-/B-cell responses with repeat dosing; preparations may activate complement and elicit anti-EV antibodies	Requires formal immunological monitoring (donor-specific antibodies, complement markers, cytokine profiles) before clinical adoption
Cost considerations	Good Manufacturing Practice (GMP) cell-banking of parental MSC line, qualified xeno-free media, TFF + size-exclusion chromatography purification, and full release-QC panel (sterility, endotoxin, NTA, marker profile, identity, potency)	On a per-patient basis, current EV manufacturing costs are comparable to autologous cell therapy and unlikely to be cost-competitive with symptomatic xerostomia therapies until allogeneic off-the-shelf products mature
Manufacturing/regulatory framework (expanded)	No harmonised potency assay tied to a defined mechanism of action; no harmonised reference standards for particle quantification; FDA places most EV products under cell-and-gene-therapy frameworks while EMA takes a case-by-case position	Regulatory ambiguity is currently the dominant barrier to first-in-human xerostomia trials, even where biological/preclinical data are supportive

## Data Availability

No new data were created or analyzed in this study. Data sharing is not applicable to this article.

## Supplementary Materials

The following supporting information can be downloaded at https://www.mdpi.com/article/10.3390/ijms27135926/s1.
